# Supplementary material for: One Hundred Consecutive Neutropenic Febrile Episodes Demonstrate That CXCR3 Ligands Have Predictive Value in Discriminating the Severity of Infection in Children with Cancer
Source: Children (Basel). 2022 Dec 25;10(1):39. doi: 10.3390/children10010039 (PMC9857223; doi:10.3390/children10010039)
Supplement: Supplementary file 1 [file children-10-00039-s001.zip › Table S1.pdf]

Table S1. Type of infection in Group B and Group C.

Type of infection	Pathogen	Number of episodes	ICU admission
Group B (n=47)			
Gastroenteritis microbiologically documented	Escherichia coli ESBL (+)<br>Klebsiella pneumonia ESBL (+)<br>Klebsiella pneumoniae ESBL (+); Escherichia<br>coli ESBL (+)<br>Enterobacter cloacae ESBL (+)<br>Enterobacter spp. ESBL (+)<br>Salmonella spp.<br>Citrobacter freundii ESBL (+)<br>Stenotrophomonas maltophilia	8<br>2<br>1<br>1<br>1<br>1<br>1<br>1	0<br>0<br>0<br>0<br>0<br>0<br>0<br>0
Gastroenteritis clinically documented	Not detected	6	0
Mucositis	Not detected	8	0
Pneumonia	Not detected	7	0
Urinary tract infection	Enterobacter cloacae ESBL (+)<br>Escherichia coli ESBL (+)<br>Klebsiella oxytoca ESBL (-)<br>Klebsiella pneumoniae ESBL (-)	2<br>2<br>1<br>1	0<br>0<br>0<br>0
Soft tissue infections	Not detected	4	0
Group C (n=19)			
Bacteriemia:<br>Gram-positive<br><br><br><br><br><br>Gram-negative	 Staphylococcus epidermidis MRCNS<br>Staphylococcus haemolyticus—MRCNS<br>Staphylococcus aureus MSSA<br>Staphylococcus chromogenes MSCNS<br>Staphylococcus hominis—MRCNS<br><br>Enterobacter cloacae ESBL (+)<br>Enterobacter cloacae ESBL (-)<br>Moraxella catarrhalis	 3<br>2<br>1<br>1<br>1<br><br>1<br>1<br>1	 0<br>0<br>0<br>0<br>0<br><br>0<br>0<br>0
Sepsis:<br>Gram-negative	 Enterobacter cloacae ESBL (+)<br>Pseudomonas aeruginosa	 1<br>1	 1<br>0
Septic shock clinically diagnosed	Not detected	2	1
Thyphlitis	Not detected	2	2
Severe infection of unknown cause/multiorgan failure	Not detected	2	2

MRCNS – methicyllin-resistant Staphylococcus aureus
MSSA – methicyllin-sensitive Staphylococcus aureus
ESBL – extended-spectrum beta-lactamase
ICU – Intensive Care Unit
