# Supplementary material for: One Hundred Consecutive Neutropenic Febrile Episodes Demonstrate That CXCR3 Ligands Have Predictive Value in Discriminating the Severity of Infection in Children with Cancer
Source: Children (Basel). 2022 Dec 25;10(1):39. doi: 10.3390/children10010039 (PMC9857223; doi:10.3390/children10010039)
Supplement: Supplementary file 1 [file children-10-00039-s001.zip › Table S2.pdf]

Table S2. Laboratory parameters in infection episodes.

**Parameter:**	Group A	Group B	Group C	p value	Group B+C	p value
CRP 1 (mg/dL)	16.20 (5.9-39.5)	26.1 (8.1-42.0)	24.4 (6.1-45.5)	0.48	25.65 (8.1-42.0)	0.25
PCT 1 (mg/mL)	0.16 (0.07-0.22)	0.16 (0.10-0.28)	0.24 (0.14-0.72)	0.05	0.165 (0.11-0.31)	0.15
I-TAK 1 (pg/ml)	48.635 (30.22-84.38)	71.77 (41.6-132.73)	65.12 (38.75-118.62)	0.08	70.99 (40.25-132.03)	0.026
IP-10 1 (pg/ml)	59.95 (46.45-111.8)	97.95 (58.52-291.70)	80.77 (46.62-231.38)	0.09	96.845 (56.28-246.36)	0.044
MIG 1 (pg/ml)	75.395 (57.77-184.87)	91.15 (54.57-177.91)	76.36 (43.36-315.23)	0.65	87.065 (52.52-242.99)	0.54
CRP 2 (mg/dL)	44.60 (10.5-78.9)	36.6 (18.9-84.7)	39.70 (23.4-94.5)	0.56	39.15 (21.9-85.7)	0.31
PCT 2 (mg/mL)	0.17 (0.08-0.46)	0.20 (0.11-0.38)	1.20 (0.22-3.04)	0.004	0.225 (0.14-1.10)	0.14
I-TAK 2 (pg/ml)	52.92 (31.38-101.52)	73.33 (41.6-166.35)	59.97 (35.37-122.20)	0.23	66.15 (40.25-136.26)	0.16
IP-10 2 (pg/ml)	102.40 (67.52-193.65)	168.13 (100.49-363.0)	117.58 (66.64-191.61)	0.07	149.385 (92.06-362.81)	0.046
MIG 2 (pg/ml)	81.28 (60.76-132.03)	100.21 (69.98-217.53)	72.00 (49.46-395.96)	0.46	93.225 (63.53-235.77)	0.56
