# Supplementary material for: One Hundred Consecutive Neutropenic Febrile Episodes Demonstrate That CXCR3 Ligands Have Predictive Value in Discriminating the Severity of Infection in Children with Cancer
Source: Children (Basel). 2022 Dec 25;10(1):39. doi: 10.3390/children10010039 (PMC9857223; doi:10.3390/children10010039)
Supplement: Supplementary file 1 [file children-10-00039-s001.zip › Table S3.pdf]

Table S3. The sensitivity, specificity, AUC (area under curve), SE (standard error), 95% confidence interval (CI) limits of AUC and accuracy for each parameter at cut-off values between Group A and B+C based on Youden's Index.

	Cut-off value (based on Youden's Index)	Sensitivity	Specificity	AUC	SE	Lower limit of 95% CI of AUC	Upper limit of 95% CI of AUC	P value	Accuracy
CRP 1	25.2	0.515	0.676	0.57	0.059	0.454	0.687	0.2369	0.570
PCT 1	0.13	0.727	0.455	0.59	0.062	0.469	0.711	0.1464	0.636
I-TAC 1	33.34	0.879	0.412	0.637	0.06	0.519	0.754	0.0226	0.720
IP-10 1	63.3	0.727	0.559	0.624	0.058	0.51	0.738	0.0334	0.670
MIG 1	361.33	0.197	0.971	0.538	0.058	0.424	0.652	0.5179	0.384
CRP 2	10.9	0.924	0.265	0.563	0.063	0.439	0.686	0.3212	0.700
PCT 2	0.16	0.712	0.500	0.591	0.062	0.469	0.713	0.1445	0.640
I-TAC 2	34.43	0.848	0.353	0.587	0.062	0.464	0.709	0.1649	0.680
IP-10 2	129.9	0.576	0.706	0.623	0.058	0.509	0.737	0.0348	0.620
MIG 2	217.53	0.273	0.882	0.536	0.058	0.423	0.65	0.5308	0.480
