# Supplementary material for: One Hundred Consecutive Neutropenic Febrile Episodes Demonstrate That CXCR3 Ligands Have Predictive Value in Discriminating the Severity of Infection in Children with Cancer
Source: Children (Basel). 2022 Dec 25;10(1):39. doi: 10.3390/children10010039 (PMC9857223; doi:10.3390/children10010039)
Supplement: Supplementary file 1 [file children-10-00039-s001.zip › Table S4.pdf]

Table S4. Predictive measures of the effect for determined cut-offs between Group A and B+C.

	I-TAC 1	IP-10 1	IP-10 2
Cut-off	33.34	63.3	129.9
Positive predictive value (PPV)	0.744	0.762	0.792
Negative predictive value (NPV)	0.636	0.514	0.462
Population risk	0.660	0.660	0.660
Absolute risk reduction (ARR)	0.380	0.275	0.253
Number needed to treat (NNT)	2.632	3.631	3.949
Relative risk (RR)	2.045	1.566	1.470
Lower limit of 95% confidence interval for relative risk	0.850	0.796	0.786
Upper limit of 95% confidence interval for relative risk	4.919	3.082	2.750
Odds for positive (ODDS)	2.900	3.200	3.800
Odds for negative (ODDS)	1.750	1.056	0.857
Odds ratio (OR)	5.075	3.378	3.257
Lower limit of 95% confidence interval for odds ratio	1.855	1.419	1.345
Upper limit of 95% confidence interval for odds ratio	13.884	8.039	7.889
Accuracy (ACC)	0.720	0.670	0.620
Sensitivity	0.879	0.727	0.576
Specificity	0.412	0.559	0.706
Positive likelihood-ratio (LR+)	1.494	1.648	1.958
Negative likelihood-ratio (LR-)	0.294	0.488	0.601
Youden’s Index	0.291	0.286	0.282
