# Supplementary figures and images for: One Hundred Consecutive Neutropenic Febrile Episodes Demonstrate That CXCR3 Ligands Have Predictive Value in Discriminating the Severity of Infection in Children with Cancer
Source: Children (Basel). 2022 Dec 25;10(1):39. doi: 10.3390/children10010039 (PMC9857223; doi:10.3390/children10010039)

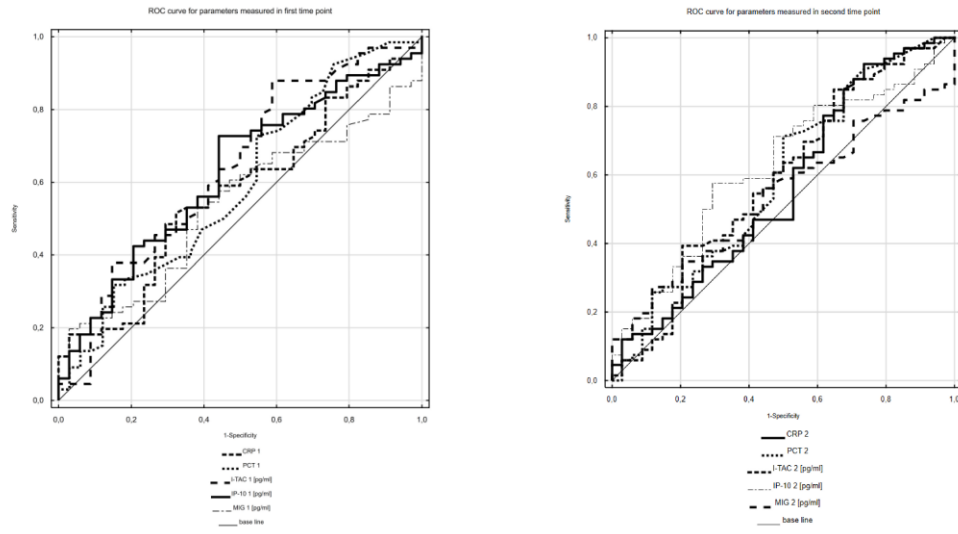

**Figure S1.** ROC curves for all analyzed parameters at both time points between Group A and B+C.

Supplement: Supplementary file 1 [file children-10-00039-s001.zip › Figure S1.pdf]
